# Interactive segmentation of membrane and membrane-mimic densities in cryo-EM maps

**DOI:** 10.1107/S205979832600598X

**Published:** 2026-07-09

**Authors:** Alok Bharadwaj, Lotte Veerbeek, Arjen Jakobi

**Affiliations:** a Department of Bionanoscience, Kavli Institute of Nanoscience Delft University of Technology 2629 HZDelft The Netherlands; University of Glasgow, United Kingdom

**Keywords:** cryo-EM, segmentation, membranes

## Abstract

Contextual density arising from biological membranes or membrane mimics such as detergent micelles or lipid nanodiscs is ubiquitous in cryo-EM maps of integral and peripheral membrane proteins, or membrane-associated complexes, from single-particle analysis and subtomogram averaging. Here, we describe *SURFER*, a lightweight, GPU-accelerated extension for *UCSF ChimeraX* that enables rapid semantic differentiation of membrane or membrane-mimic density from macromolecular signal. *SURFER* operates on unfiltered maps to automatically generate segmentation masks that can be flexibly applied to any aligned target map, including reference volumes for local refinement or post-processed maps optimized for visualization and interpretation. By allowing contextual density to be selectively toggled or subtracted, *SURFER* enables direct interactive comparison between representations with and without membrane-related features to facilitate consistent analysis and reporting of membrane context in cryo-EM structures. An interactive graphical interface within *ChimeraX* supports convenient threshold adjustment, connectivity filtering and immediate visual evaluation of the resulting segmentation.

## Abstract 2

*SURFER* performs automated segmentation of contextual membrane and membrane-mimic density in cryo-EM maps to enable robust separation of macromolecular signal from surrounding detergent or lipid–membrane features. It is conveniently distributed as a plugin for *UCSF ChimeraX*, allowing interactive application within standard map-visualization workflows.

## 1. Introduction

Cryogenic electron microscopy (cryo-EM) and electron tomography (cryo-ET) now routinely yield three-dimensional electrostatic potential maps for membrane proteins and membrane-associated complexes across a wide resolution range (Nygaard *et al.*, 2020 ▸; Chien *et al.*, 2025 ▸). Beyond the ordered macromolecular core, many cryo-EM structures of membrane proteins contain contextual density originating from sample preparation or the native environment, including detergent micelles (Privé, 2007 ▸; Hoffmann *et al.*, 2025 ▸), amphipols (Tribet *et al.*, 1996 ▸), lipid nanodiscs (Nath *et al.*, 2007 ▸; Frauenfeld *et al.*, 2016 ▸), membrane bilayers (Dietrich *et al.*, 2024 ▸) and other partially occupied or conformationally heterogeneous components (Chien *et al.*, 2025 ▸). Such contextual density is typically weakly ordered and dominated by low-resolution signal. Although it can provide important information on the organization of the membrane environment and the positioning of transmembrane regions, its incoherent signal frequently causes the density to fragment into discontinuous regions that complicate visualization and segmentation using simple threshold-based approaches. Map post-processing has a strong influence on how such contextual density is represented. Conventional global sharpening (Rosenthal & Henderson, 2003 ▸) and many map-enhancement approaches (Sanchez-Garcia *et al.*, 2021 ▸; Terwilliger *et al.*, 2020 ▸; He *et al.*, 2023 ▸; Selvaraj *et al.*, 2024 ▸) prioritize the recovery of high-resolution contrast in well ordered regions that often form the bulk of the macromolecular signal. While effective for enhancing macromolecular features, these approaches often further fragment low-resolution density or suppress it altogether (Hoffmann *et al.*, 2016 ▸; Berkeley *et al.*, 2024 ▸). In contrast, map post-processing strategies that aim to preserve local signal statistics can retain contextual density at intensity levels comparable to those of the macromolecule (Jakobi *et al.*, 2017 ▸; Ramlaul *et al.*, 2019 ▸; Bharadwaj *et al.*, 2025 ▸). Building on these principles, *LocScale*-2.0 was introduced to improve the interpretability of cryo-EM maps through confidence-guided map optimization while maintaining a more unbiased representation of both ordered and disordered components (Bharadwaj *et al.*, 2025 ▸). As a result, membrane and membrane-mimic density often appears more continuous and interpretable in *LocScale*-2.0-optimized maps at a common intensity threshold.

While retention of contextual density is advantageous for assessing membrane topology and macromolecular environment, it also introduces challenges during interactive map inspection and visual presentation. In particular, lipid or detergent density may obstruct the view on transmembrane regions or dominate visualization at commonly used contour levels. This creates a practical need for tools that allow contextual density to be selectively segmented and interactively visualized. In addition to visual presentation, segmentation of membrane-mimic density can be useful during 3D refinement. From an image-formation perspective, a cryo-EM micrograph represents a tomographic projection of the specimen modulated by the point-spread function of the electron microscope (Singer & Sigworth, 2020 ▸). When membrane proteins are embedded in a lipid bilayer, nanodisc or detergent micelle, these components can produce a strong contribution to the projection signal, which in some orientations may exceed that of the embedded protein itself. The strong low-resolution signal of detergent micelles or lipid nanodiscs, where detergent and lipid molecules are expected to adopt unrelated positions away from the immediate environment of the protein, can negatively impact iterative refinement procedures that assume a largely uniform signal, and can lead to overfitting. Although data-driven regularization strategies such as non-uniform refinement (Punjani *et al.*, 2020 ▸) and local signal filtering (Ramlaul *et al.*, 2020 ▸) reduce sensitivity to disordered regions, selective masking and focused refinement workflows remain widely used in practice for restricting alignment and reconstruction to regions of interest or for removing nontarget signal by subtraction (Liu & Sigworth, 2014 ▸; Jensen *et al.*, 2016 ▸; Ilca *et al.*, 2015 ▸; Bai *et al.*, 2015 ▸; Fernández-Giménez *et al.*, 2021 ▸).

Segmentation of cryo-EM density maps has traditionally relied on intensity-based thresholding (Otsu, 1979 ▸), morphological watershed approaches (Najman & Schmitt, 1994 ▸; Volkmann, 2002 ▸) and region-based methods that combine connectivity with multi-scale smoothing or statistical merging (Pintilie *et al.*, 2010 ▸; Nock & Nielsen, 2004 ▸). These approaches form the basis of widely used tools such as *Segger* and related workflows for partitioning macromolecular assemblies into compact subregions (Pintilie *et al.*, 2010 ▸). More recent methods incorporate additional constraints, for example graph-based representations and symmetry information (Terashi *et al.*, 2020 ▸). While effective for separating well defined macromolecular components, these methods are primarily designed to identify regions characterized by relatively sharp intensity gradients or compact connectivity. Lipid and detergent-associated density, by contrast, is typically smooth, weakly bounded and sometimes spatially contiguous with the macromolecular envelope, making its segmentation sensitive to contour levels and connectivity criteria.

**Figure 1:**
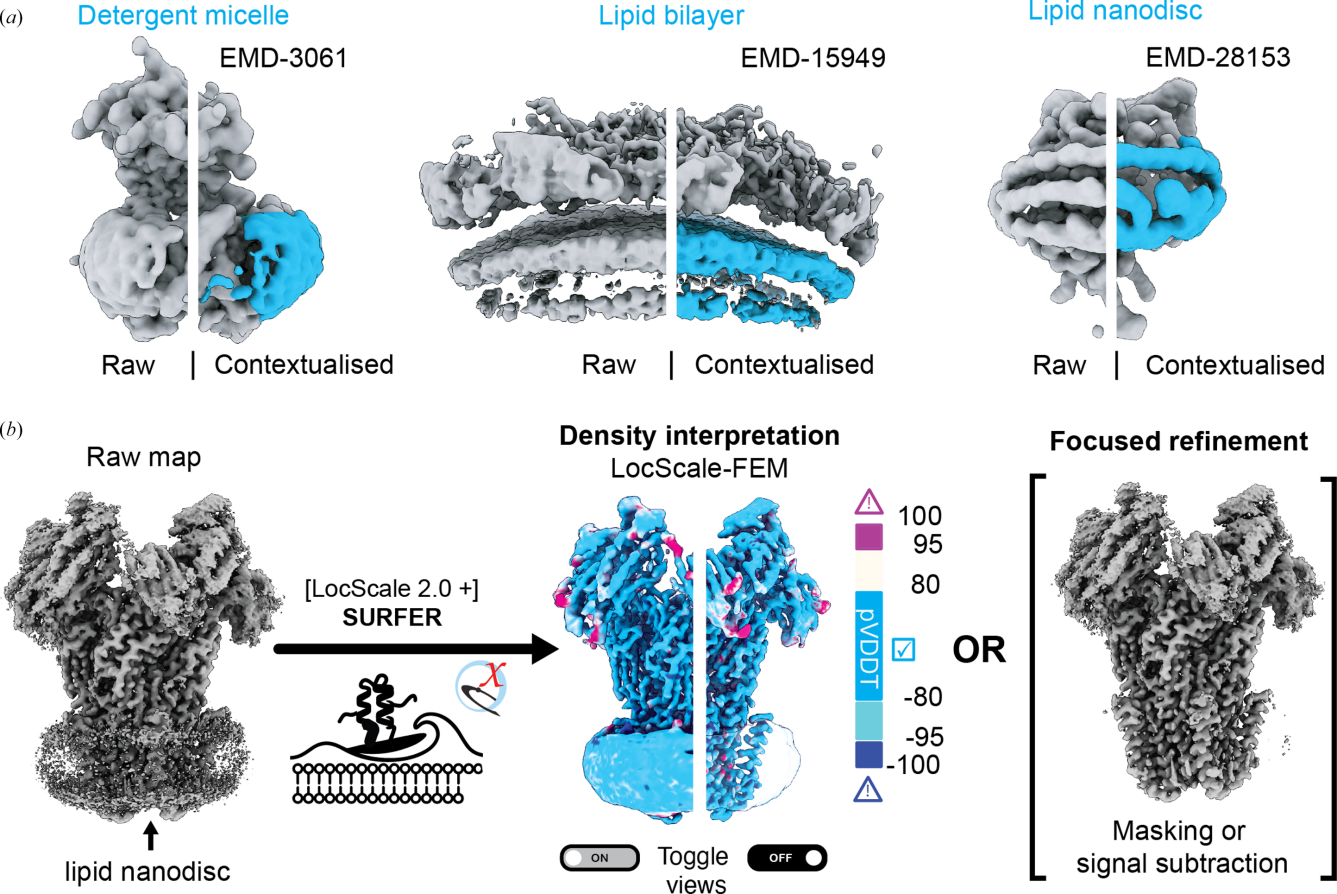
Contextualization of lipid densities and applications of *SURFER*. (*a*) Raw and contextualized cryo-EM density maps for γ-secretase in amphipol A8-35 (EMDB entry EMD-3061), COPII coat on lipid bilayers (EMDB entry EMD-15949) and the mechanosensitive channel TMEM63A in lipid nanodiscs (EMDB entry EMD-28153). (*b*) Schematic illustration of *SURFER* segmentation and subtraction of lipid micelle for visualization with *LocScale*-2.0-optimized maps or for particle subtraction for 3D refinement. The example shown is the pentameric calcium-sensitive channel DeCLIC from *Desulfofustis glycolicus* reconstituted in lipid nanodiscs (EMDB entry EMD-19995). The LocScale-FEM pVDDT confidence score is mapped onto the optimized map.

Similar challenges are well recognized in cryogenic electron tomography (cryo-ET) image analysis, where membranes are commonly segmented using dedicated methods developed for low signal to noise and diffuse boundaries, including tensor voting (Martinez-Sanchez *et al.*, 2014 ▸), deep learning-based voxel classification (Chen *et al.*, 2017 ▸; Lamm *et al.*, 2022 ▸; de Teresa-Trueba *et al.*, 2023 ▸; Morales-Martínez *et al.*, 2025 ▸) and combinations of deep learning-based segmentation with parametric fitting and interactive curation (Siggel *et al.*, 2024 ▸). Such hybrid strategies are often required to accommodate the wide range of membrane geometries encountered in practice. Comparable variability is also observed in single-particle reconstructions of membrane proteins, where contextual density may arise from membrane mimics such as micelles, bicelles, nanodiscs or planar bilayers and can differ substantially across datasets. Robust segmentation of such contextual density therefore benefits from combining prior information on membrane location with data-driven voxel classification. To aid in the analysis of contextual densities in single-particle reconstructions and subtomogram averages, we have developed *SURFER* (Segmentation of Unstructured Regions and Filtering for Enhanced Representation), a lightweight framework for machine-learning-based segmentation of lipid and detergent densities. *SURFER* automatically identifies, segments and optionally subtracts lipid and detergent densities corresponding to micelles, lipid bilayers or lipid nanodiscs, thereby allowing users to focus alternately on the macromolecular core or the assembly in its membrane context (Fig. 1 ▸). While originally designed for use in conjunction with *LocScale*-2.0 map optimization, the segmentation mask generation of *SURFER* operates on unfiltered maps and its application is therefore compatible with any raw or post-processed map.

*SURFER* is implemented as a *ChimeraX* (Meng *et al.*, 2023 ▸) bundle with both graphical user interface and command-line functionality. Here, we describe the design and training of the segmentation model, evaluate its performance across a diverse set of cryo-EM density maps from EMDB-deposited membrane-protein structures and illustrate how *SURFER* enables contextualization of lipid and membrane-mimic densities and their subtraction from macromolecular signal in cryo-EM structures.

## 2. Methods

### 2.1. Software environment

Model training and inference were implemented in PyTorch (v2.9) with *CUDA* 12.6. Data processing and analysis were performed in Python (v3.11). Cryo-EM map and atomic model operations were carried out using *EMmer* (Bharadwaj & Jakobi, 2022 ▸), a Python library for cryo-EM map and model processing built on *mrcfile* (Burnley *et al.*, 2017 ▸) and *gemmi* (Wojdyr, 2022 ▸).

### 2.2. Training dataset generation

#### 2.2.1. Selection of map–model pairs

Training data were derived from cryo-EM reconstructions containing detergent micelles or membrane-associated density. Half-map pairs for 500 EMDB entries were initially collected. From these, 254 reconstructions containing visible lipid membranes, nanodiscs or detergent belts were identified through automated filtering and manual inspection. From this initial set, only entries with corresponding atomic models deposited in the Protein Data Bank (PDB; wwwPDB Consortium, 2019 ▸) and annotated membrane orientations in the OPM database (Lomize *et al.*, 2006 ▸) were retained. After target-mask generation and manual quality control (see below), 186 map–model pairs were retained. Of these, 144 were used for training and 26 for validation (an 85:15 split), while 16 were reserved as an independent test set. The full curated set is listed in Supplementary Tables S1 and S2.

#### 2.2.2. Molecular envelope and difference mask generation

For each reconstruction in the curated training and validation set, a mask defining the molecular envelope was generated using false discovery rate (FDR)-controlled thresholding (Bharadwaj *et al.*, 2025 ▸; Beckers *et al.*, 2020 ▸). Unfiltered half maps were averaged prior to FDR estimation. Noise statistics were estimated from cubic regions extracted from the edges of the reconstruction volume. The noise box size was set to the larger of 20^3^ voxels or 10% of the box dimension of the reconstructed volume. To obtain smooth and conservative molecular boundaries, maps were low-pass filtered to 5 Å before FDR thresholding. All masks were binarized at an FDR of 1% and used consistently throughout training and validation. Density contained in the FDR mask but not explained by the atomic model was identified by subtracting a model-derived mask from the FDR mask. Model masks were generated from simulated model maps using *gemmi* (Wojdyr, 2022 ▸) by including all voxels within 3 Å of any atom in the deposited structure. The resulting difference mask frequently contained thin shell artefacts at protein boundaries due to minor discrepancies between model and envelope estimation. These artefacts were removed by uniform filtering followed by re-binarization.

#### 2.2.3. Prediction and refinement of membrane boundaries

Approximate membrane orientations were obtained from OPM (Lomize *et al.*, 2006 ▸). Because OPM coordinates are not aligned to deposited PDB models, all OPM structures were rigid-body aligned to their corresponding PDB entries using *gemmi*. Membranes were assumed to be planar for training data generation; curved membranes were excluded. Each membrane was represented by two parallel planes of the form
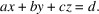
Initial plane parameters were estimated from three non-collinear points sampled from the predicted membrane surface. To refine the plane positions, edges in the difference mask were enhanced using a Sobel filter. Slices were extracted along the direction normal to the membrane planes, and the number of foreground voxels per slice was computed to generate an intensity profile. Peaks in the gradient of this profile were used to determine the refined membrane boundaries. The difference mask was subsequently cropped between the two refined planes.

#### 2.2.4. Geometric characterization of contextual density

To quantify geometric variability across detergent- and membrane-associated densities, two complementary descriptors were computed from binarized contextual volumes: a flatness ratio and a fragmentation ratio. Volumes were binarized at a threshold of 0.5 prior to feature extraction.

##### 2.2.4.1. Flatness ratio

The flatness ratio provides a measure of the aspect ratio of the micelle or membrane envelope. A best-fitting ellipsoid was computed for each binarized volume using *pyradiomics* (van Griethuysen *et al.*, 2017 ▸). The height of the micelle was defined as the minor principal axis of the ellipsoid and the width as the major principal axis. The flatness ratio was defined as
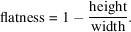
Values approaching 1.0 indicate highly flattened, bilayer-like geometries, whereas lower values correspond to more isotropic or weakly anisotropic detergent belts.

##### 2.2.4.2. Fragmentation ratio

Spatial continuity of the segmented density was quantified using a surface-area-to-volume metric that describes the degree of fragmentation. Binarized volumes were converted to triangular meshes using the marching cubes algorithm (Lorensen & Cline, 1987 ▸). The surface area was computed as the sum of triangle areas, and the enclosed volume was obtained by tetrahedral decomposition. The fragmentation ratio was defined as
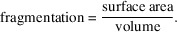
Because area and volume are computed in physical units using the map pixel size, this ratio has units of Å^−1^. Higher values indicate increased roughness or fragmentation.

### 2.3. Connectivity filtering

A binary segmentation sometimes contains multiple mutually disconnected regions, including unrelated low-resolution density. Connected-component analysis was performed using the label function implemented in *scikit-image* and only the largest connected component was retained. The filtered volumes were manually inspected for dataset curation. Entries for which micelle density could not be reliably isolated were excluded. This curation step yielded the final dataset used for training, validation and testing.

### 2.4. Segmentation network

#### 2.4.1. Network architecture

*SURFER* employs a 3D Swin-Conv U-Net (SCUNet; Zhang *et al.*, 2023 ▸). The encoder consists of three blocks, each composed of a Swin-Conv module followed by strided convolution for downsampling. Within each Swin-Conv module, window-based transformer layers operate in parallel with convolutional layers and their outputs are concatenated prior to resolution reduction. A single Swin-Conv module forms the bottleneck. The decoder mirrors the encoder using transposed convolutions for upsampling and skip connections to corresponding encoder features. Details are summarized in Supplementary Table S3 and Fig. S2(*a*).

#### 2.4.2. Pre-processing and augmentation

All input maps were resampled to 1 Å per voxel and intensities were standardized to zero mean and a standard deviation of 0.1. Data augmentation was applied prior to cube extraction and included random rotations about Cartesian axes, translations up to ±10 voxels, *B*-factor modulation (random shifts between 0 and 400 Å^2^; applied twice per map) and Gaussian blurring corresponding to effective resolutions between 5 and 20 Å.

#### 2.4.3. Subvolume sampling

Training samples consisted of cubic subvolumes of size 48 Å. Cubes were extracted on a regular Cartesian grid with 26 Å spacing. Grid positions were classified as signal or noise using the resampled FDR mask. All signal-containing cubes were retained. Additional noise cubes were randomly sampled to achieve a signal-to-noise cube ratio of 4:1.

#### 2.4.4. Training parameters

The network was trained using cube-wise binary cross-entropy (BCE) loss with L1 regularization and the Adam optimizer with default momentum parameters (β = [0.9, 0.999]). Training was performed over eight epochs. The optimal weights for the L1 regularizer, learning rate and batch size were determined empirically using the Optuna optimization framework (Akiba *et al.*, 2019 ▸). The optimal values were found to be as follows: L1 weight decay of 0.0025, a learning rate of 5 × 10^−4^ and a batch size of 64.

### 2.5. *ChimeraX* integration

*SURFER* is distributed as a *ChimeraX* bundle and can be installed via the ChimeraX Toolshed or from the source repository. In *ChimeraX*, the tool is accessible under Tools → Volume Data. The bundle includes all required metadata and trained model weights within the source distribution. The workflow comprises two stages. Firstly, unfiltered half maps are supplied for segmentation. If preferred, a user-defined mask can also be provided. Secondly, the predicted segmentation is applied to any aligned target map. Users select a binarization threshold to define the final segmentation mask, which may be optionally smoothed using a uniform filter with a default width of five voxels. Contextual density can be interactively toggled to compare representations with and without membrane signal.

## 3. Results

### 3.1. Overview

Our research was motivated by a lack of accessible tools specifically optimized for segmentation of membrane or detergent micelle-related densities in three-dimensional (3D) electrostatic potential maps from cryo-EM single-particle analysis and subtomogram averaging. *SURFER* is a tool designed to fill this gap (Fig. 1 ▸*a*). The method uses voxel-wise classification with a hybrid convolutional–transformer architecture to distinguish contextual density from the ordered macromolecular signal. Segmented contextual density can then be interactively visualized or subtracted from a target map to enable direct comparison between representations that retain or suppress membrane-related features, or for the generation of masks for focused refinement (Fig. 1 ▸*b*). In this section, we first describe the generation of a curated training dataset and the design of the segmentation model. We then evaluate segmentation performance across a diverse set of deposited cryo-EM reconstructions and analyse the geometric variability of lipid and detergent environments captured in the training data. Finally, we illustrate practical use cases of *SURFER*, including interactive visualization and subtraction of contextual density, and assess the robustness of the approach under different segmentation thresholds and filtering strategies.

### 3.2. Training dataset generation and characteristics

**Figure 2:**
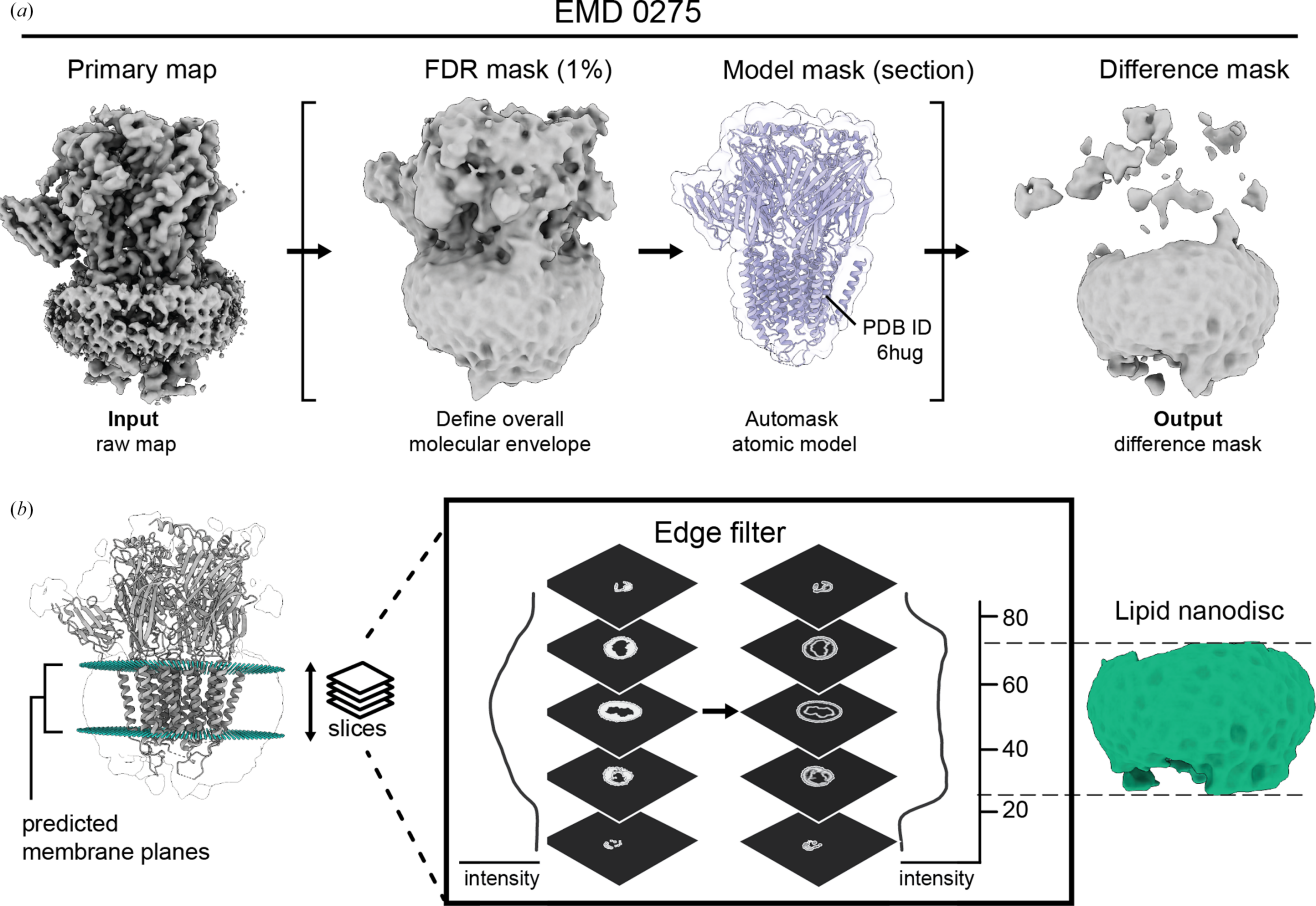
Training dataset generation for membrane/detergent segmentation. (*a*) Difference mask generation for isolation of lipid density regions illustrated with α1β3γ2L GABAA receptor in lipid nanodiscs (EMDB entry EMD-0275). Left: raw average of the unfiltered half maps from which the FDR confidence mask is generated. The ordered volume is estimated from the deposited atomic model (PDB entry 6hug) and subtracted from the FDR-enclosed volume to generate a difference mask. Note that the raw difference mask is not restricted to the detergent micelle but can encompass other unmodelled signal. (*b*) Predicted membrane planes relative to the atomic model and the raw difference mask and procedure of edge detection to refine plane positioning at the detergent boundary. The segmented detergent micelle volume is shown on the far right.

We assembled a labelled dataset of segmented lipid and detergent densities from curated map–model pairs of integral membrane and membrane-associated proteins derived from the Electron Microscopy Data Bank (EMDB; wwPDB Consortium, 2024 ▸), restricted to entries with associated atomic models encompassing the full transmembrane region. To isolate disordered lipid and detergent density, we generated difference masks by determining the molecular envelope via statistical thresholding with false discovery rate (FDR) control (Bharadwaj & Jakobi, 2022 ▸; Bharadwaj *et al.*, 2025 ▸; Beckers *et al.*, 2020 ▸) and subtracting the ordered volume occupied by the atomic coordinate model. The resulting masks contain density that is not explained by the atomic model, including lipid or detergent-related density, together with other unmodelled structural features (Fig. 2 ▸*a*). We then further limited this volume to membrane or membrane-mimic density by predicting the planes circumscribing micelles, lipid nanodiscs or bilayer membranes around the transmembrane region using *Positioning of Proteins in Membranes* 3.0 (*PPM* 3.0), which positions membrane proteins by minimizing the free energy of transfer into an anisotropic solvent model (Lomize *et al.*, 2011 ▸, 2022 ▸). While *PPM* 3.0 supports both planar and curved membranes, we restricted analyses here to planar geometries and reserve curved membranes for future work. Comparison of the predicted boundaries to the cryo-EM data revealed that *PPM* 3.0 systematically underestimated lipid-bilayer and micelle thickness. To correct this, we refined boundary positions by computing intensity gradients normal to the predicted planes after enhancing intensity transitions with a Sobel filter (Fig. 2 ▸*b*). By scanning through the volume along the plane normal, the locations of maximal ascending and descending gradients can then be identified and used to define the final membrane or micelle boundaries.

**Figure 3:**
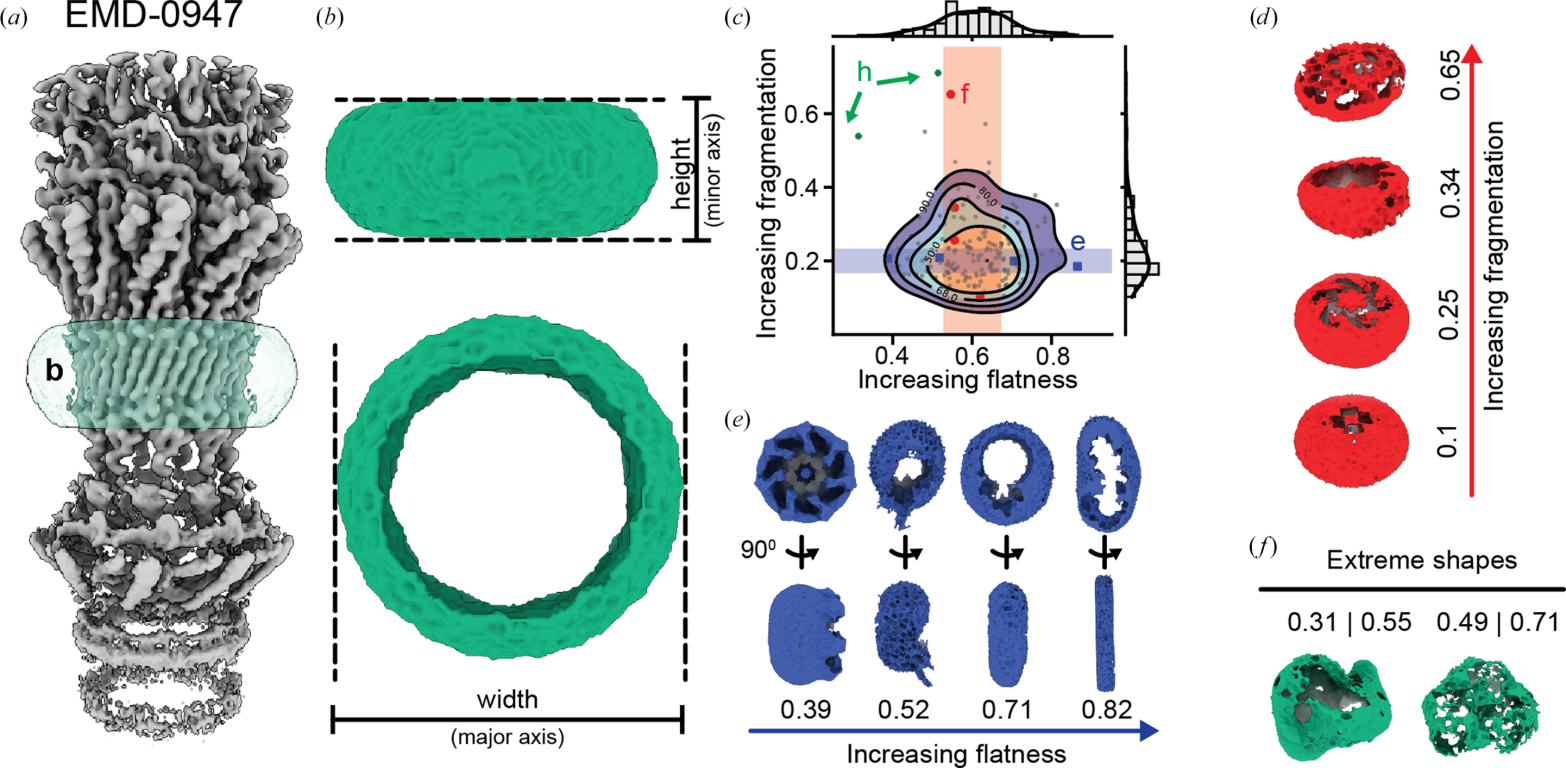
Geometric variability of membrane-mimic density in the training dataset. (*a*, *b*) Geometrical properties of segmented micelle volume shown for the enterobacterial curli secretion complex CsgG–CsgF (EMDB entry EMD-0947). (*c*) Distribution of shape features from the training dataset (*N* = 170). Semi-transparent rectangles denote bands of largest variation along fragmentation and flatness ratios, respectively. (*d*, *e*) Representative micelle densities contained within the high-variability bands. (*f*) Out-of-distribution outliers marked by arrows in (*c*). Numbers indicate flatness and fragmentation ratio, respectively.

We applied this workflow to extract lipid and detergent densities from the averaged half maps of 254 sequence-diverse membrane-protein structures deposited in the EMDB, of which 186 high-quality segmentations were retained after curation (Fig. 2 ▸*b*, Supplementary Fig. S1). In most cases, these densities appear smooth because the majority of lipids or detergent molecules in membranes, micelles or nanodiscs are mobile, resulting in averaging out of structural detail such that no individual lipid or detergent molecules can be identified. This typically means that the micelle, nanodisc or membrane components in these complexes are disordered beyond a resolution of about 10–20 Å (Figs. 3 ▸*a* and 3 ▸*b*). The shape of these lipid or detergent structures is highly pleomorphic, depending on both the shape and symmetry of the embedded membrane protein or complex and the properties and composition of the surrounding micelle or membrane (Supplementary Fig. S1). We characterized this variability by measuring flatness and fragmentation levels across the micelle dataset (Figs. 3 ▸*c*–3 ▸*f*). The variation in flatness reflects the different physicochemical properties and molecular-packing geometry of the detergent or lipid molecules in micelles, bicelles and nanodiscs. Fragmentation likely arises from low signal-to-noise ratios in the raw data. Estimating molecular boundaries when signal strength approaches noise levels is inherently challenging and can produce discontinuous, rough surfaces. While some micelles exhibit uniform fragmentation in all directions, others display anisotropy, potentially reflecting preferential protein–lipid interactions along specific orientations. Together, the flatness and fragmentation distributions shown in Fig. 3 ▸(*c*) provide a compact representation of the geometric variability captured in the training dataset. For a newly analysed membrane-protein structure, projecting the segmented contextual density into this feature space offers a simple way to assess whether its morphology falls within the range represented during training. Membrane mimics whose geometric descriptors lie within the main density of the training distribution are expected to be well supported by the learned model (see Section 2.3), whereas pronounced outliers may provide a practical indication that a given membrane geometry is underrepresented in the training data and could constitute a more challenging, potentially out-of-distribution case.

### 3.3. Segmentation model and performance

We used the annotated dataset to train a 3D Swin-Conv-UNet (SCUNet) for voxel-wise segmentation of cryo-EM density maps (Supplementary Fig. S1). The network follows a U-Net-style encoder–decoder architecture with skip connections (Ronneberger *et al.*, 2015 ▸) and is composed of Swin-Conv blocks that combine convolutional processing with window-based vision-transformer modules (Liang *et al.*, 2021 ▸; Zhang *et al.*, 2023 ▸). Within each block, convolutional layers capture local density features, while transformer blocks aggregate information within fixed-size spatial windows. Although SCUNet was originally introduced for image-restoration tasks (Zhang *et al.*, 2023 ▸), we adapt the same principle here for binary voxel-wise classification of lipid membrane or detergent micelle density versus macromolecular signal, as the combination of convolutional layers and shifted window-based transformer modules enable a straightforward method for context aggregation of spatially extended density features (Park & Kim, 2022 ▸). Details of the network architecture, training protocol and optimization behaviour are summarized in Supplementary Table S3, Supplementary Figs. S2(*a*)–S2(*d*) and described in Section 2.

### 3.4. Threshold dependence and connectivity filtering

**Figure 4:**
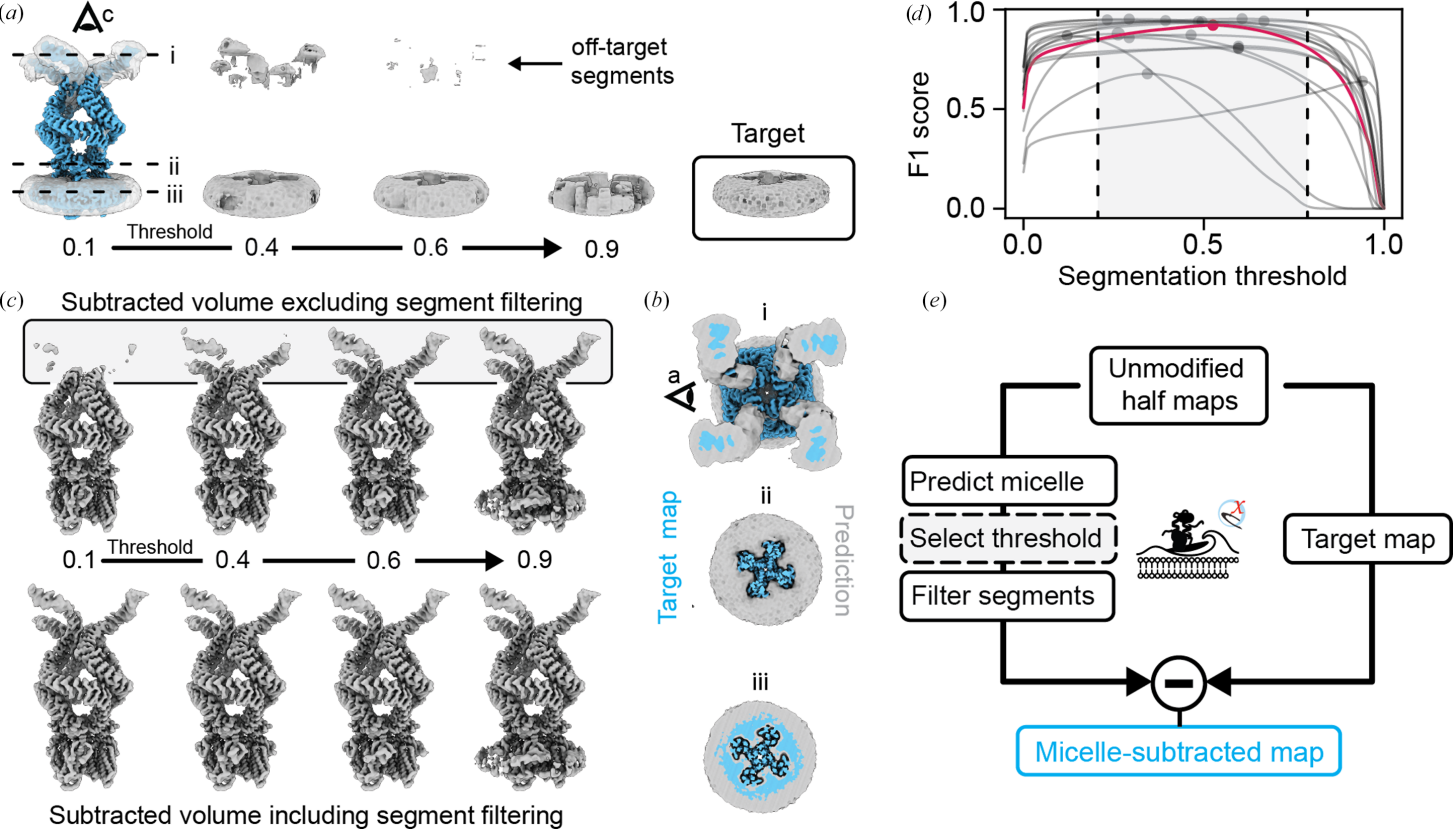
Threshold dependence and connectivity filtering. (*a*) Illustration of off-target segments in membrane-distal regions of nanodisc-embedded *D. melanogaster* NOMPC (EMDB entry EMD-8702). Density contours for different binarization thresholds are shown. (*b*) 2D section views at the three section planes indicated in (*a*) show the overlap of the unfiltered micelle prediction with the target map. (*c*) Effect of filtering based on largest connected volume on the micelle-subtracted volume. The top row shows how subtraction of the predicted segment without filtering also removes density from the off-target segments. The bottom row shows how segment filtering restricts subtraction to only the micelle density. (*d*) F1 score as a function of binarization threshold evaluated on the held-out test dataset. Segmentation performance peaks at an intermediate threshold, reflecting the trade-off between recall at low thresholds and precision at high thresholds. The position and sharpness of this optimum vary across reconstructions. The curve for EMDB entry EMD-8702 is shown in red. (*e*) Schematic illustration of the interactive thresholding workflow implemented in *SURFER*, highlighting how users can explore the effect of threshold choice on segmentation quality and apply connectivity filtering prior to density subtraction.

*SURFER* produces a continuous-valued voxel-wise confidence score indicating the likelihood that a given voxel belongs to membrane or membrane-mimic density. To obtain a segmentation mask for visualization or density subtraction, this output must be binarized at a chosen threshold. Because membrane-mimic density typically occupies only a small fraction of the molecular volume and is dominated by weakly bounded signal, *SURFER* produces probabilistic outputs with rather broad confidence distributions near the class boundaries (Supplementary Fig. S2*e*). As a result, converting these probabilities into binary masks is sensitive to the chosen threshold, and the threshold that yields optimal segmentation can vary across datasets. At low segmentation thresholds, this sensitivity can lead to false-positive inclusion of low-resolution density that is not part of the membrane or membrane mimic. A good example illustrating this effect is the nanodisc-embedded transient receptor potential (TRP) ion channel NOMPC (Jin *et al.*, 2017 ▸). NOMPC contains 29 ankyrin-repeat domains (AR^1–29^), of which AR^1–7^ are highly flexible and resolved only at low resolution comparable to that of the surrounding lipid nanodisc. As shown in Figs. 4 ▸(*a*) and 4 ▸(*b*), *SURFER*-generated segmentation masks at low thresholds include substantial off-target density corresponding to these flexible AR^1–7^ domains. Increasing the threshold progressively suppresses this off-target signal; however, rather aggressive thresholds are required before it is fully eliminated, at which point the nanodisc density itself begins to fragment and thus results in incomplete subtraction (Fig. 4 ▸*c*). A practical way to alleviate this trade-off is to exploit the characteristic connectivity of membrane and membrane-mimic density. Such density typically forms extended and spatially contiguous regions, whereas most off-target density will mainly be composed of smaller and disconnected components. *SURFER* therefore includes an optional connectivity filtering step in which only the largest connected component of the binarized segmentation mask is retained prior to subtraction. As illustrated in Fig. 4 ▸(*c*), including this connectivity filter can help exclude spurious off-target density while preserving the membrane-mimic envelope at moderate thresholds, and thus allow clean subtraction of the lipid nanodisc.

To more generally examine the effect of binarization threshold on segmentation quality, we evaluated voxel-wise segmentation accuracy as a function of threshold using the F1 score, computed on the held-out test dataset for which 16 curated reference segmentations were available. The F1 score, defined as the harmonic mean of precision and recall, provides a measure of segmentation performance that is particularly informative in the presence of strong class imbalance, as is the case here (Supplementary Fig. S2*d*). As shown in Fig. 4 ▸(*d*), the F1 score exhibits a pronounced dependence on the chosen segmentation threshold. For any given reconstruction, there exists a threshold at which the best balance is achieved between recovering the membrane-mimic density and avoiding false-positive inclusion of unrelated low-resolution signal. At low thresholds recall is high, but precision is reduced due to the inclusion of off-target low-resolution signal, leading to moderate overall performance. Increasing the threshold suppresses these false positives and improves precision, resulting in a peak F1 score at an intermediate threshold. At still higher thresholds, recall decreases as the predicted membrane-mimic density becomes fragmented or incomplete, again reducing the F1 score. The location and shape of this optimum vary between reconstructions, but for most reconstructions F1 scores are relatively flat across a range of segmentation thresholds centred on 0.5. This behaviour highlights two points. Firstly, even though a generic 0.5 threshold appears well suited as a reasonable best guess, segmentation quality can probably not be reliably optimized using a single global threshold across datasets. Secondly, the threshold dependence observed in the quantitative evaluation mirrors the practical challenges encountered during visual inspection, where users may have to balance removal of smooth contextual density against the preservation of weak but structurally relevant features. These observations motivate the interactive threshold selection and connectivity-based filtering implemented in *SURFER*, which allow users to adapt the segmentation to the specific characteristics of each reconstruction rather than relying on a fixed global threshold (Fig. 4 ▸*e* and Supplementary Fig. S2*f*).

### 3.5. Interactive segmentation and context exploration

**Figure 5:**
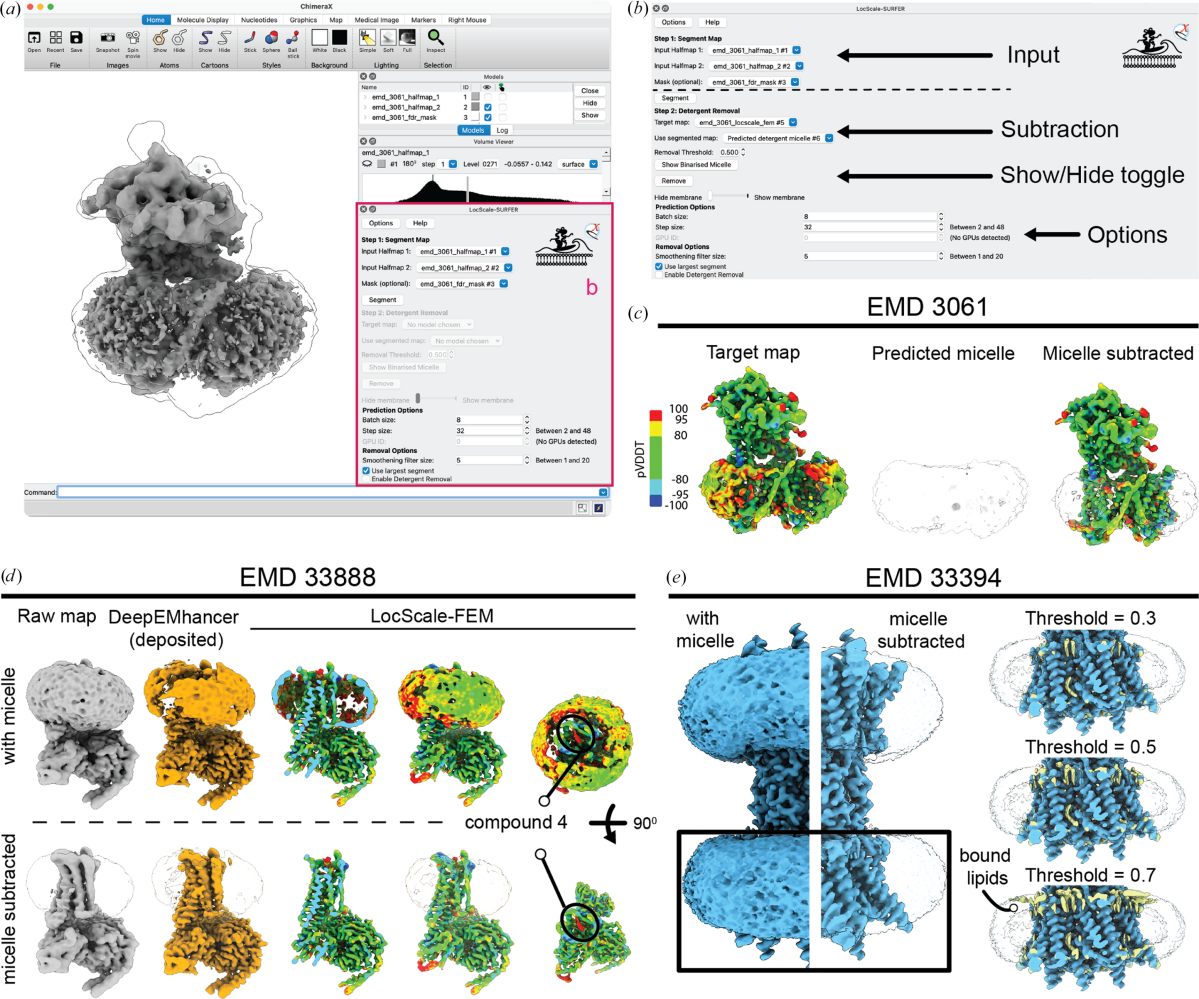
Interactive segmentation and contextual density control with *SURFER* in *UCSF ChimeraX*. (*a*) *ChimeraX* interface illustrating *SURFER* applied to the γ-secretase complex (EMDB entry EMD-3061). The averaged unfiltered map is shown in grey, with the outline of the FDR-based confidence mask superposed. (*b*) Close-up of the *SURFER* tool interface. *SURFER* accepts a pair of unfiltered half maps as input and optionally a molecular boundary mask. Predicted micelle density can be visualized across a range of binarization thresholds. Once a threshold is selected, the segmented micelle can be subtracted from any target map aligned to the raw input. In this example, a LocScale-FEM optimized map (Bharadwaj *et al.*, 2025 ▸) is used as the target. (*c*) LocScale-FEM target map, *SURFER*-predicted micelle density and the corresponding micelle-subtracted map for EMDB entry EMD-3061. (*d*) Application of *SURFER* to the relaxin family peptide receptor 4 (EMDB entry EMD-33888). Shown are the raw map, the deposited *DeepEMhancer*-optimized map and the LocScale-FEM map before (top) and after (bottom) micelle subtraction. Density corresponding to the bound RXFP4 ligand is preserved in the subtracted map. (*e*) Application of *SURFER* to the connexin43 (Cx43/GJA1) gap-junction intercellular channel (EMDB entry EMD-33394). A *LocScale*-2.0-optimized target map is shown before and after micelle subtraction. Density associated with tightly bound lipids is retained in the micelle-subtracted map but its retention depends on the chosen binarization threshold.

*SURFER* is deployed as an interactive plugin within the *UCSF ChimeraX* environment (Fig. 5 ▸*a*). *ChimeraX* is increasingly used for atomic coordinate and density-map display and manipulation, making it a natural platform for a user-friendly contextual segmentation workflow. The plugin provides a *ChimeraX* interface to the *SURFER* tool, enabling users to perform contextual segmentation directly during map inspection and analysis (Fig. 5 ▸*b*). However, all *SURFER* functions can also be programmatically accessed via the command line if desired. The workflow comprises an automated segmentation step followed by interactive threshold refinement and application. The *SURFER* tools allows users to specify a pair of unfiltered, independent half maps as input. From these, *SURFER* computes an FDR-controlled confidence mask to define the molecular boundary and subsequently predicts detergent- or membrane-associated density within this region. The predicted contextual density can be interactively thresholded to generate a binary segmentation mask, which can then be applied to any target map aligned with the input to selectively remove micelle or membrane signal (Fig. 5 ▸*b*). Target maps may include the unfiltered input map itself, for example to generate masks for local 3D refinement, or post-processed maps intended for display and figure generation, such as contextualized maps produced by *LocScale*-2.0 FEM map optimization. The *ChimeraX* interface allows users to toggle between representations with and without contextual density to facilitate direct visual comparison. Depending on the map size, the procedure typically completes within seconds for smaller reconstructions and within approximately 10 min for the largest maps tested (Table 1 ▸).

Fig. 5 ▸(*c*) illustrates this workflow for the human γ-secretase complex (EMDB entry EMD-3061; Bai *et al.*, 2015 ▸). In this example, the target map is a *LocScale*-2.0 feature-enhanced map (LocScale-FEM), in which ordered macromolecular regions and more poorly resolved contextual density including the detergent micelle are represented on a comparable intensity scale. Application of *SURFER* segmentation to the LocScale-FEM map enables selective removal of the surrounding micelle while preserving the protein-associated transmembrane features that benefit from *LocScale*-2.0 optimization such as the TMD2 (Fig. 5 ▸*c*). We also tested the ability of the segmentation workflow in distinguishing smooth contextual density arising from mobile detergent molecules or lipids from density corresponding to ordered lipids or small-molecule ligands at the protein–micelle interface. Fig. 5 ▸(*d*) shows the result for the relaxin family peptide receptor 4 (RXFP4; EMDB entry EMD-33888), which contains a weakly bound amidrazone scaffold compound located at the transmembrane region (Bharadwaj *et al.*, 2025 ▸; Chen *et al.*, 2023 ▸). We performed micelle subtraction on the raw reconstruction, the author-deposited *DeepEMhancer*-optimized map and a LocScale-FEM map of RXFP4. Comparison of the maps before and after micelle subtraction shows that in this case *SURFER* effectively removes the surrounding micelle while preserving the ligand density at the automatically determined segmentation threshold. There are, however, cases in which the segmentation mask at the default threshold may encroach upon less well ordered density in transmembrane regions. Fig. 5 ▸(*e*) illustrates the application of *SURFER* to the hexameric connexin43 (Cx43/GJA1) gap-junction intercellular channel (EMDB entry EMD-33394) in lipid nanodiscs, again using a *LocScale*-2.0-optimized target map (Bharadwaj *et al.*, 2025 ▸; Lee *et al.*, 2023 ▸). The Cx43/GJA1 structure of EMDB entry EMD-33394 (PDB entry 7xqf) contains 24 modelled copies of the cholesterol ester cholesteryl hemisuccinate (CHS) and 12 copies of the phospholipid 1-palmitoyl-2-oleoyl-phosphatidylethanolamine (POPE) tightly associated with the transmembrane domains of both hemichannels. When binarizing the segmentation mask at the default threshold of 0.5 or lower, in this case, the retention of ordered lipid features depends on the chosen binarization threshold for the segmentation mask, reflecting their partial spatial overlap with the segmented contextual density. It thus appears that interactive threshold adjustment can be necessary to balance the suppresion of smooth contextual density and the retention of structured, protein-associated lipid density.

### 3.6. Generalization across membrane mimics and geometries

**Figure 6:**
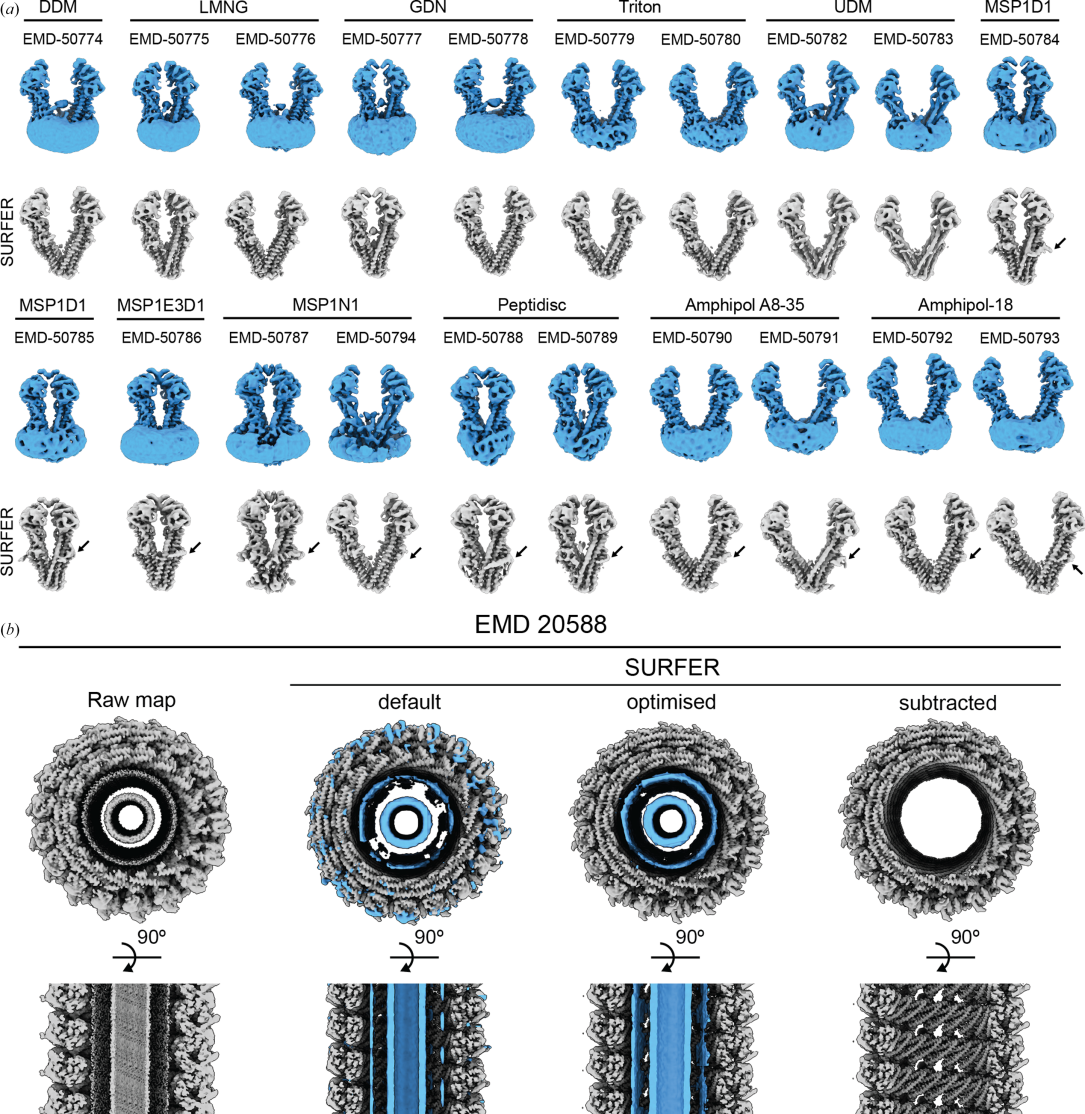
Segmentation and subtraction of diverse membrane mimics and curved membranes. (*a*) LocScale-FEM maps of MsbA solved in 12 different membrane mimics (EMDB entries EMD-50774 to EMD-50794) before (blue) and after (grey) segmentation and micelle subtraction with *SURFER*. Arrows indicate retained density of membrane-scaffold proteins, peptidiscs and amphipols. (*b*) Segmentation of curved membrane tubes for EMDB entry EMD-20588. *SURFER* segmentations are shown for default settings and after optimization; the subtracted map corresponds to the optimized segmentation

To assess the ability of *SURFER* to segment contextual density across a broad range of membrane environments, we investigated a set of 20 cryo-EM structures of the bacterial ATP-binding cassette transporter MsbA determined in 12 distinct membrane-mimetic systems (EMDB entries EMD-50774 to EMD-50794). This dataset provides a controlled benchmark in which the macromolecular core adopts a limited set of conformations, while the surrounding contextual density varies substantially in geometry, intensity and other characteristics. The series includes classical detergents (DDM, LMNG, GDN, Triton and UDM), nanodiscs assembled with different membrane-scaffold proteins (MSP) variants, peptidiscs and amphipols (Hoffmann *et al.*, 2025 ▸). These membrane mimics produce contextual density ranging from compact, approximately spherical micelles to flattened nanodiscs and irregular peptidisc belts (Fig. 6 ▸*a*). We applied *SURFER* segmentation to all 20 reconstructions using the default binarization threshold and subtracted the predicted lipid or detergent density from each map (Fig. 6 ▸*a*). Across the set of 20 maps, *SURFER* consistently identified the dominant detergent or lipid envelope surrounding MsbA despite pronounced differences in micelle shape and thickness. Differences in segmentation quality appear to primarily be associated with variations in signal continuity and resolution of the membrane-mimic density rather than systematic misclassification. Subtraction of the predicted contextual density preserved the structured transmembrane helices and cytosolic domains of MsbA across the series. In several nanodisc and amphipol reconstructions, density corresponding to membrane-scaffold proteins or polymer belts remained partially visible (arrows in Fig. 6 ▸*a*). These results indicate that *SURFER* generalizes across a broad range of experimentally distinct membrane-mimetic systems without mimic-specific parameter adjustment and captures shared geometric and intensity features of contextual lipid and detergent density. While we noted the current limitations for curved membrane geometries in Section 2.2, we also tested *SURFER* on strongly curved lipid tubes such as those induced by the membrane-bound ESCRT-III filament composed of CHMP1B and IST1 (EMDB entry EMD-20588). *SURFER* segmentation with default settings appeared to capture the membrane density well, although it also included some low-resolution protein density at the filament periphery. Optimized thresholding and application of a Laplacian filter to the input map enabled reliable segmentation and subtraction of the membrane tube in this and other examples (Fig. 6 ▸*b*, Supplementary Fig. S3*a*). This suggests that the current *SURFER* model may be more sensitive to the intensity gradients and contrast differences between weakly ordered contextual lipid density and well ordered protein density than to membrane geometry *per se*. Other curved membrane examples showed less consistent behaviour with incomplete membrane segmentation and/or the inclusion of nonmembrane density (Supplementary Figs. S3*b*–S3*d*). These differences likely reflect map-specific combinations of local contrast, membrane continuity and overlap between low-resolution protein and lipid density, in addition to the limited representation of curved membrane geometries in the current training data. Thus, segmentation of curved membrane geometries still requires further optimization and validation, and the primary use case for the current implementation remains micelle and membrane-mimic subtraction in single-particle cryo-EM maps.

### 3.7. Generalization to intermediate and low-resolution density maps

**Figure 7:**
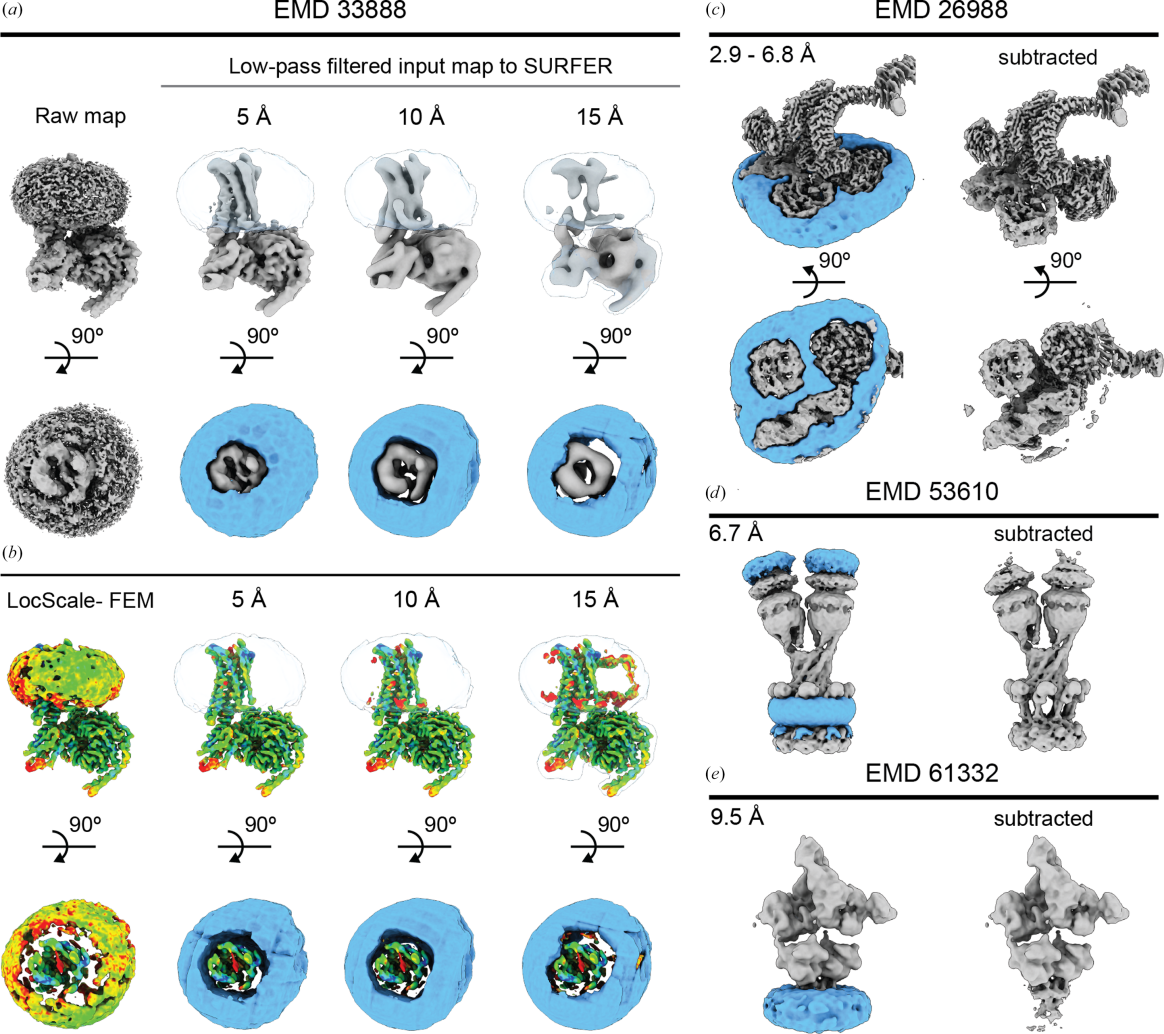
Generalization to mid- and low-resolution maps. (*a*) Micelle subtraction for relaxin family peptide receptor 4 (EMDB entry EMD-33888) after the application of *SURFER* to maps low-pass filtered to progressively lower resolution. (*b*) Micelle subtraction from the LocScale-FEM map of EMDB entry EMD-33888 using segmentation masks generated from the input maps shown in (*a*). (*c*, *d*, *e*) Representative applications to additional mid- and low-resolution maps: EMDB entries EMD-26988, EMD-53610 and EMD-61332 shown before segmentation and micelle subtraction on the left and after subtraction on the right. The micelle density segmented by *SURFER* is shown in blue in all panels.

To further test whether *SURFER* is applicable beyond high-resolution reconstructions, we evaluated segmentation performance after progressively low-pass filtering input maps and on independent EMDB entries spanning intermediate to low resolution. We first performed an artificial test case in which the RXFP4 map (EMDB entry EMD-33888) was low-pass filtered to 5, 10 and 15 Å before micelle prediction with *SURFER*. The resulting masks broadly preserved the overall boundary between the surrounding contextual density and the molecular density of the enclosed transmembrane region, although at very low resolution the boundary between micelle and protein density became increasingly blurred, reducing the accuracy of subtraction (Fig. 7 ▸*a*). This effect appears to arise primarily from blurring of the protein density itself, as application of the same segmentation masks to the high-resolution LocScale-FEM density of EMDB entry EMD-33888 showed that the masks do not substantially cut into well resolved protein density. We next tested *SURFER* on more challenging experimental maps (Fig. 7 ▸*b*). EMDB entry EMD-26988 provides a particularly informative example because it represents a composite map of the native human erythrocyte ankyrin-1 complex containing an asymmetric membrane-associated assembly with components resolved to different levels of detail. Despite this heterogeneity, *SURFER* identified the surrounding micelle or membrane-mimetic density and enabled its subtraction while retaining all of the macromolecular density (Fig. 7 ▸*c*). We also applied *SURFER* to EMDB entry EMD-53610, a 6.7 Å resolution map of the Wza–Wzc complex that mediates the transport of capsular polysaccharides across the bacterial envelope and spans the inner and outer membranes of Gram-negative bacteria. In this case, *SURFER* segmented multiple membrane-associated density regions for both inner and outer membrane-embedded parts, which requires switching the connectivity filter off (Fig. 7 ▸*d*). Finally, we applied *SURFER* to EMDB entry EMD-61332, a substantially lower resolution map reported at 9.5 Å, demonstrating that segmentation remains possible even when high-resolution structural features are largely absent. These examples indicate that *SURFER* does appear to generalize reasonably well to lower resolution maps, extending its practical use to cases where manual masking is often ambiguous.

## 4. Discussion and conclusions

Automated segmentation of membrane and membrane-mimic density in cryo-EM maps presents challenges distinct from those encountered when segmenting density of ordered macromolecular components. Primarily inspired by *LocScale*-2.0 (Bharadwaj *et al.*, 2025 ▸), which optimizes map representations to emphasize contextual density, *SURFER* was built to provide automated segmentation of membrane-related signal together with interactive control over its visualization. While not explicitly tested for this purpose, the procedure may in principle facilitate mask generation for membrane subtraction or focused refinement. This could be advantageous for small integral membrane proteins, where dominant low-resolution micelle or nanodisc signal may influence alignment. However, caution is warranted as subtracting signal from particle images or reconstructions risks the removal of structurally relevant signal. While the observed robustness of *SURFER* segmentation is encouraging in this respect, systematic evaluation of its suitability for subtraction-based and focused refinement strategies remains the subject of future work.

The results presented here show that *SURFER* can reliably identify membrane-mimetic density across a wide range of membrane-protein structures and membrane-mimic geometries. In many cases, default segmentation parameters appear to be sufficient to accurately separate and subtract contextual density while preserving ordered transmembrane regions. In more challenging situations, interactive adjustment of the binarization threshold may be required to balance the removal of smooth contextual signal against the retention of structured features. The segmentation and subtraction procedure typically completes within a few minutes on a conventional laptop, making it well suited for interactive use. The integration of *SURFER* into the *UCSF ChimeraX* environment allows users to directly assess the plausibility of the segmentation, compare maps with and without contextual density, and generate masks for downstream applications without leaving the visualization environment. *SURFER* appears to generalize reasonably well across different membrane mimics but is less accurate for membrane geometries that are underrepresented in the training data, such as curved lipid bilayers. In such cases, segmentation quality is reduced and greater user intervention is required. Extending the training dataset to include a broader range of membrane geometries is therefore a natural direction for future work.

Overall, *SURFER* provides a straightforward and accessible approach for contextual density segmentation in cryo-EM maps. By enabling selective suppression or visualization of membrane and detergent density during interactive analysis, it complements existing map processing and visualization tools and facilitates more objective interpretation of membrane-protein structures.

## Supplementary Material

Supplementary Tables and Figures. DOI: 10.1107/S205979832600598X/id5015sup1.pdf

SURFER SCUNet model.: https://zenodo.org/records/15488062

## Figures and Tables

**Table 1:** *SURFER* runtime for selected EMDB entries

EMDB entry	Box size (voxels)	Pixel size (Å)	Box size (Å)	Volume (×10^6^ Å^3^)	Time
EMD-30017	240	1.03	247.2	15.1	36–38 s
EMD-3061	180	1.40	252.0	16.0	30–40 s
EMD-8702	400	1.20	480.0	110.6	3–4 min
EMD-13234 †	336	1.70	571.2	186.3	4–5 min
EMD-19999 †	460	1.63	749.8	421.5	10–12 min

† Map does not contain micelle density; used to quantify runtime only.

## Data Availability

*SURFER* is open-source software released under a BSD license and is available at https://github.com/cryotud/locscale-surfer, where detailed documentation is also provided. *SURFER* is also available directly from the ChimeraX Toolshed at https://cxtoolshed.rbvi.ucsf.edu. The *SURFER* segmentation model (v0.1) can be accessed via Zenodo (https://zenodo.org/records/15488062). Scripts used for data analysis and for the generation of figures shown in this manuscript can be found at https://github.com/cryotud/publications.
