# Supplementary material for: Interactive segmentation of membrane and membrane-mimic densities in cryo-EM maps
Source: Acta Crystallogr D Struct Biol. 2026 Jul 9;82(Pt 8):848–61. doi: 10.1107/S205979832600598X (PMC13431642; doi:10.1107/S205979832600598X)
Supplement: Supplementary file 1 [file d-82-00848-sup1.pdf]

STRUCTURAL BIOLOGY

Volume 82 (2026)

Supporting information for article:

Interactive segmentation of membrane and membrane-mimic densities in cryo-EM maps

Alok Bharadwaj, Lotte Veerbeek and Arjen Jakobi

Table S1. EMDB and PDB identifiers for maps and models used in SURFER training and validation set.

#	EMDB	PDB	#	EMDB	PDB	#	EMDB	PDB	#	EMDB	PDB	#	EMDB	PDB	#	EMDB	PDB	#	EMDB	PDB
1	23276	7lck	2	31399	7f0j	3	20244	6p46	4	10933	6yup	5	13882	7qb9	6	22316	7jga	7	24958	7sax
8	9932	6k7h	9	10830	6ykr	10	9112	6mgv	11	11799	7ain	12	22311	7jg5	13	24825	7s3i	14	32110	7vsg
15	31433	7f3f	16	10995	6yxr	17	13035	7oqz	18	10919	6ytl	19	22979	7koo	20	8354	5t4d	21	0976	6lto
22	21453	6vxz	23	30904	7dxc	24	11720	7ad3	25	22120	6xbm	26	22376	7jlp	27	23425	7lll	28	9935	6k7j
29	21972	6wzg	30	12142	7bcq	31	0200	6hd1	32	4270	6fkf	33	21705	6wkn	34	0926	6ln7	35	23436	7lly
36	13008	7ooa	37	21234	6vm0	38	0924	6ln5	39	10828	6ykm	40	30713	7dlu	41	9941	6k7m	42	25492	7sx3
43	25691	7t4x	44	30535	7d0i	45	30907	7dxf	46	13155	7p14	47	9695	6iok	48	0827	6l3v	49	8118	5irz
50	11733	7adp	51	22983	7kox	52	30017	6lyp	53	10184	6sgt	54	13985	7qia	55	0925	6ln6	56	12939	7oj8
57	22689	7k65	58	11782	7ahc	59	22882	7ki0	60	0928	6ln9	61	4789	6rb9	62	23476	7lpc	63	4733	6r69
64	20278	6p9y	65	11208	6zgj	66	24956	7sat	67	13095	7ow8	68	4611	6qp6	69	13662	7puy	70	0919	6lmt
71	30219	7bvg	72	21512	6w1m	73	4587	6qm4	74	20194	6ot5	75	11588	6zzx	76	12026	7b5d	77	22264	6xmu
78	20537	6pzt	79	31010	7e84	80	10049	6rx4	81	4411	6i53	82	20488	6pv8	83	7099	6bhu	84	30903	7dxb
85	22771	7kai	86	30216	7bvc	87	25800	7tbw	88	21236	6vm2	89	4594	6qmb	90	10094	6s3q	91	9937	6k7k
92	0947	6lqj	93	21672	6whg	94	25803	7tc0	95	23093	7kzz	96	20508	6pwn	97	10895	6ysf	98	22772	7kaj
99	9696	6iol	100	9119	6mhv	101	0469	6np0	102	21511	6w1j	103	0275	6hug	104	9113	6mgw	105	0975	6ltn
106	13201	7p5m	107	0921	6lmv	108	13969	7qhb	109	0501	6nt4	110	10735	6y9b	111	0094	6gyo	112	0280	6huk
113	9942	6k7n	114	4645	6qv6	115	26188	7tyn	116	9851	6jnf	117	9931	6k7g	118	24957	7sau	119	10182	6sgr
120	0915	6lly	121	0488	6nr3	122	21518	6w1y	123	0487	6nr2	124	8117	5irx	125	21671	6whc	126	21237	6vm3
127	12095	7b93	128	4919	6rld	129	30906	7dxe	130	0004	6giq	131	20509	6pwo	132	3860	5oyb	133	0923	6lmx
134	0199	6hcy	135	10185	6sgu	136	0716	6kls	137	22375	7jlo	138	30217	7bve	139	0498	6nsj	140	10829	6ykp
141	23473	7lp9	142	0922	6lmw	143	24310	7r87	144	7039	6b3j	145	4592	6qm9	146	30575	7d4p	147	31135	7egk
148	30576	7d4q	149	0499	6nsk	150	13880	7qa8	151	11629	7a46	152	30908	7dxg	153	12296	7nf6	154	30218	7bvf
155	6822	6jpf	156	4972	6roh	157	0981	6lum	158	4593	6qma	159	12996	7onj	160	0093	6gyn	161	4612	6qpb
162	0912	6lle	163	0927	6ln8	164	9610	6adq	165	31003	7e7s	166	20145	6oo4	167	20715	6ubt	168	20143	6oo3
169	6770	5xsy	170	9115	6mho															

Table S2. EMDB and PDB identifiers for maps and models used in SURFER test set.

#	EMDB	PDB	#	EMDB	PDB	#	EMDB	PDB	#	EMDB	PDB	#	EMDB	PDB	#	EMDB	PDB	#	EMDB	PDB
1	0257	6hra	2	4032	5lc5	3	4272	6fki	4	4288	6fo2	5	4588	6qm5	6	4589	6qm6	7	4646	6qvb
8	4746	6r7x	9	7009	6ave	10	7133	6bqv	11	7882	6dg7	12	8702	5vkq	13	8958	6e1n	14	8960	6e1p
15	9934	6k7i	16	9939	6k7l															

Table S3. SCUNet architecture summary

Layer (name)	Input	Output	InC	OutC	Params	Kernel
SCUNet	48^3	48^3	1	1	–	–
Head	48^3	48^3	1	32	–	–
Conv3d	48^3	48^3	1	32	864	3^3
Down-sampling block 1	48^3	24^3	32	64	–	–
ConvTransBlock (0)	48^3	48^3	32	32	19,469	–
ConvTransBlock (1)	48^3	48^3	32	32	19,469	–
Conv3d (2)	48^3	24^3	32	64	16,384	2^3
Down-sampling block 2	24^3	12^3	64	128	–	–
ConvTransBlock (0)	24^3	24^3	64	64	76,826	–
ConvTransBlock (1)	24^3	24^3	64	64	76,826	–
Conv3d (2)	24^3	12^3	64	128	65,536	2^3
Down-sampling block 3	12^3	6^3	128	256	–	–
ConvTransBlock (0)	12^3	12^3	128	128	305,204	–
ConvTransBlock (1)	12^3	12^3	128	128	305,204	–
Conv3d (2)	12^3	6^3	128	256	262,144	2^3
Bottleneck	6^3	6^3	256	256	–	–
ConvTransBlock (0)	6^3	6^3	256	256	1,216,616	–
ConvTransBlock (1)	6^3	6^3	256	256	1,216,616	–
Up-sampling block 3	6^3	12^3	256	128	–	–
ConvTranspose3d (0)	6^3	12^3	256	128	262,144	2^3
ConvTransBlock (1)	12^3	12^3	128	128	305,204	–
ConvTransBlock (2)	12^3	12^3	128	128	305,204	–
Up-sampling block 2	12^3	24^3	128	64	–	–
ConvTranspose3d (0)	12^3	24^3	128	64	65,536	2^3
ConvTransBlock (1)	24^3	24^3	64	64	76,826	–
ConvTransBlock (2)	24^3	24^3	64	64	76,826	–
Up-sampling block 1	24^3	48^3	64	32	–	–
ConvTranspose3d (0)	24^3	48^3	64	32	16,384	2^3
ConvTransBlock (1)	48^3	48^3	32	32	19,469	–
ConvTransBlock (2)	48^3	48^3	32	32	19,469	–
Tail	48^3	48^3	32	1	–	–
Conv3d (0)	48^3	48^3	32	1	864	3^3
**Total parameters:** 4,729,084		**Trainable:** 4,727,676			**Non-trainable:** 1,408	

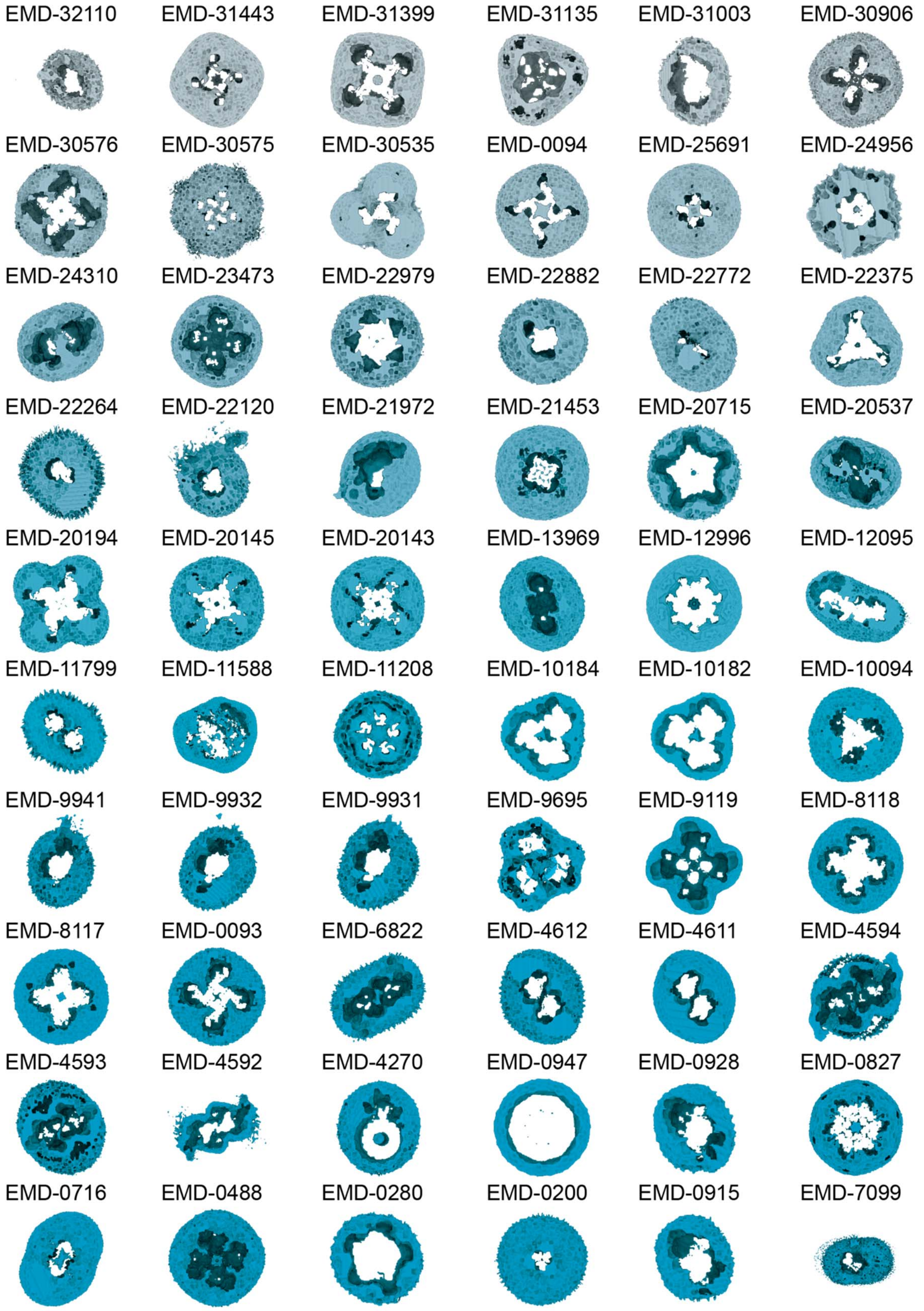

Figure S1 Shape variety of segmented membrane mimic densities. | Randomly selected examples of segmented membrane mimic densities in the training data.

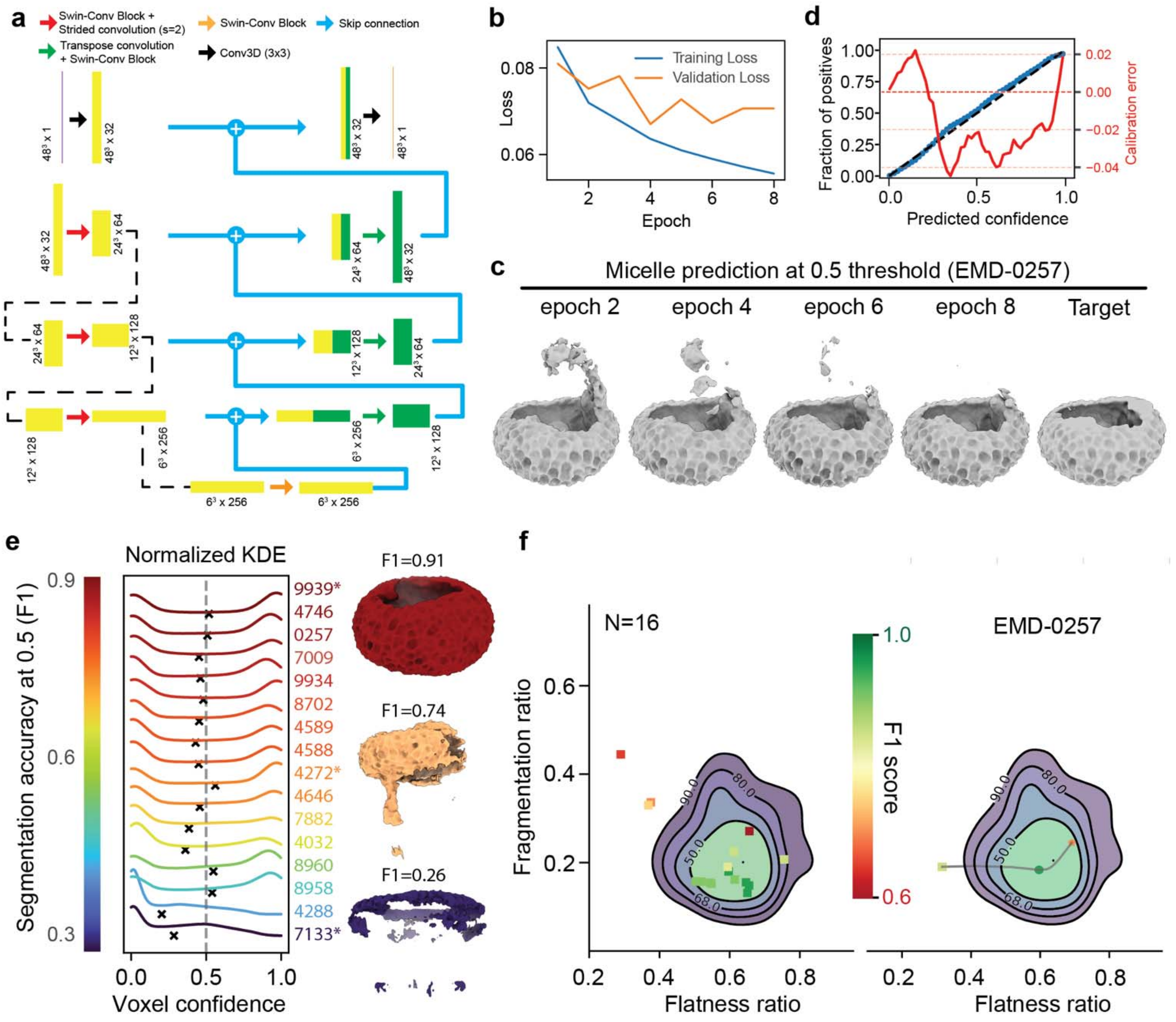

Figure S2 | Network architecture, training behaviour and confidence characteristics of SURFER predictions. | (a) Network architecture of the SURFER SCU-Net. (b) Training and validation loss curves over eight epochs, showing stable optimisation without divergence between training and validation loss. (c) Predicted detergent micelle segmentation for a held-out test map (EMD-0257) using model checkpoints from successive epochs, binarised at a fixed threshold of 0.5. The overall micelle geometry is recovered early during training, while continued optimisation primarily reduces false-positive regions and sharpens the micelle boundary at the same threshold. (d) Reliability diagram aggregating voxel-wise predictions across all test maps, showing predicted confidence versus the observed fraction of true positives. The red curve indicates calibration error (predicted confidence minus observed fraction), with negative values corresponding to under-confidence. (e) Kernel density estimates of voxel-wise predicted confidence for individual test maps, weighted by the relative micelle volume within the molecular boundary. High-accuracy segmentations (top) show clearly bimodal confidence distributions with peaks near 0 and

1, whereas lower-accuracy cases (bottom) exhibit a skew toward low-confidence values, reflecting bias toward the majority class. Representative segmentations binarised at a threshold of 0.5 are shown alongside each distribution, with F1 scores indicated. (f) Left: Fragmentation and flatness ratios of the 16 test maps, thresholded at their respective optimal F1 scores, superimposed on the flatness–fragmentation distribution of the SURFER training set (see Figure 3c). Right: Trajectory of one representative test map (EMD-0257) across different binarisation thresholds (square: 0.1; circle: 0.5; asterisk: 0.9). Data points are coloured according to F1 score.

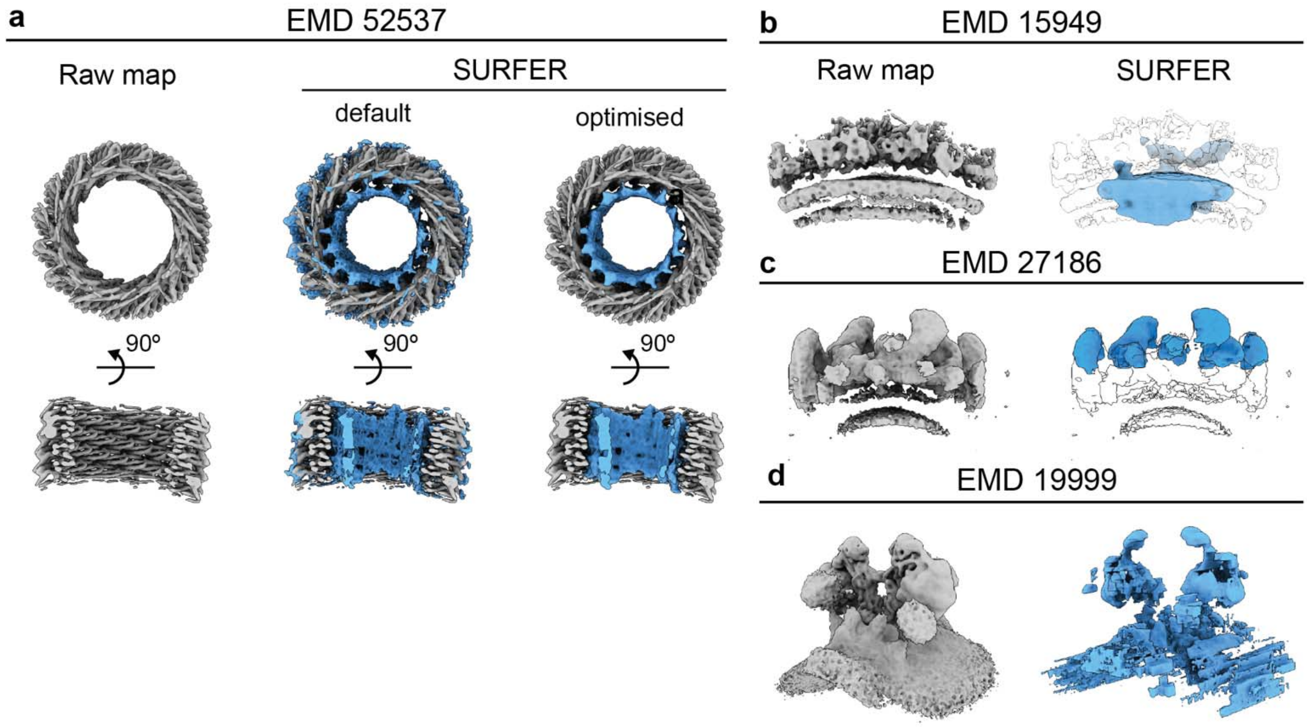

Figure S3 |Generalisation to curved membranes**.** | (a) SURFER segmentation of membrane-assembled PspA tubes from *Synechocystis* sp. PCC 6803 (EMDB: EMD-52537) with default settings and after optmisation. (b-d) Problematic cases for SURFER segmentation of curved membranes for the COPII inner coat (EMDB: EMD-15949) , AP1 lattice (EMDB: EMD-27186) and *Polytomella* mitochondrial ATP synthase (EMDB: EMD-19999).
